# Proteomic Analysis of Protein Ubiquitination Events in Dairy Goats with Fatty Liver

**DOI:** 10.3390/ani15142010

**Published:** 2025-07-08

**Authors:** Yuli Zhu, Zhenhua Liu, Yuming Zhang, Yao Meng, Xunuo Song, Jinyu Li, Yue Zhang, Junkang Zhao, Liyin Du, Qinghua Deng

**Affiliations:** 1 College of Animal Science and Technology, Inner Mongolia Minzu University, Tongliao 028000, China; m18735479882@163.com (Y.Z.); 15647402116@163.com (Z.L.); zhangyuming1117@163.com (Y.Z.); 18047698649@163.com (Y.M.); 19827937636@163.com (X.S.); lee0470@yeah.net (J.L.); 13948540952@163.com (Y.Z.); zjk20152022@163.com (J.Z.); 2 Inner Mongolia Engineering Technology Research Center of Prevention and Control the Beef Cattle Disease, Tongliao 028000, China; 3 Beef Cattle Industry School of Inner Mongolia, Tongliao 028000, China

**Keywords:** fatty liver, dairy goat, proteomics, ubiquitination

## Abstract

Fatty liver is a major metabolic disease in periparturient dairy goats. Protein ubiquitination, a type of dynamic and multifaceted post-translational modification, plays an important role in metabolism by regulating the stability and function of target proteins. However, the hepatic protein ubiquitination profile in dairy goats with fatty liver is yet to be elucidated. In this study, we collected liver and blood samples from healthy dairy goats (Con, *n* = 3) and dairy goats with fatty liver (FL, *n* = 3). Then, we analyzed the overall ubiquitination of hepatic proteins in dairy goats with fatty liver through quantitative ubiquitin label-free proteomics and bioinformatics. Proteins showing significantly altered levels of ubiquitination were identified via bioinformatics, and related regulatory pathways were screened. The results showed that the blood levels of beta-hydroxybutyric acid and non-esterified fatty acids were significantly upregulated in dairy goats with fatty liver, and a total of 238 ubiquitination sites across 921 proteins were found to be differentially altered in the fatty liver group. Among them, ubiquitination was upregulated at 351 sites across 93 proteins and downregulated at 570 sites across 145 proteins. In addition, GO and KEGG pathway analysis revealed that the differentially ubiquitinated proteins were enriched in pathways regulating lipid metabolism, such as the PPAR signaling pathway, fatty acid degradation, and peroxisome activity. Notably, by observing the overlap among these three sub-networks, we found that proteins with downregulated ubiquitination—such as ACSL1, ACSL5, EHHADH, and ACAA1—were transcriptionally upregulated in dairy goats with fatty liver. This study reveals the key ubiquitinated proteins in dairy goats with fatty liver and provides a more comprehensive understanding of the pathogenesis of fatty liver in dairy goats.

## Simple Summary

This study examined the hepatic protein ubiquitination profiles of periparturient dairy goats with fatty liver, a major metabolic disorder. Liver and blood samples were collected from healthy goats (Con, *n* = 3) and goats with fatty liver (FL, *n* = 3) and subjected to quantitative proteomics and bioinformatics analysis. FL goats exhibited elevated serum levels of β-hydroxybutyric acid (BHBA) and non-esterified fatty acids (NEFA), and histological analysis revealed extensive vacuolar degeneration and higher triglyceride levels in their hepatic tissues. In total, 238 differentially altered ubiquitination sites were identified across 921 proteins in FL goats, with 351 sites being upregulated and 570 sites being downregulated. Gene Ontology (GO) and Kyoto Encyclopedia of Genes and Genomes (KEGG) analyses showed that these proteins were enriched in lipid metabolism pathways, including PPAR signaling, fatty acid degradation, and peroxisome activity. Notably, proteins with downregulated ubiquitination (e.g., ACSL1, ACSL5, EHHADH, ACAA1) were transcriptionally upregulated in FL goats. These results highlight the critical role of ubiquitination in the pathogenesis of fatty liver and offer insights for future research on metabolic regulation in dairy goats.

## 1. Introduction

Goat milk contains smaller particles of lactose and is abundant in adenosine triphosphate (ATP), which effectively decomposes the lactose in goat milk. Therefore, goat milk is less likely to cause allergic reactions than cow milk and may be suitable for individuals with lactose intolerance [1]. Some studies show that goat milk lipoglobulin provides stronger immune-boosting capabilities than cow milk [2]. In recent years, the nutritional value of goat milk has become more widely recognized, and attention has shifted toward metabolic diseases, such as fatty liver disease, in high-yielding dairy goats [3,4,5].

The liver is a major organ that regulates the balance of carbohydrate, fat, and protein metabolism in the body [6,7,8]. In ruminants, balanced energy metabolism is particularly crucial during the perinatal period, and the liver serves as the primary site of energy metabolism and detoxification in these animals [9,10,11]. Fatty liver is a highly prevalent periparturient disease in ruminants, and is typically caused by a combination of obesity, stress, and a negative energy balance [12]. This condition is associated with decreased productivity, impaired reproductive performance, weakened liver function, and reduced milk production [13]. Moreover, it increases the risk of infectious diseases such as mastitis and uterine infections [14]. In periparturient dairy cows, fatty liver leads to a decline in milk production, shortened lifespans, prolonged calving intervals, and reduced reproductive efficiency, causing significant economic losses for dairy farms [15,16]. Although the pathogenesis of fatty liver in dairy cows has been explored considerably in studies from China and the rest of the world, reports on this condition in periparturient dairy goats remain limited. Understanding the mechanisms underlying fatty liver progression could enable the development of effective treatment and prevention strategies, potentially minimizing economic losses and safeguarding animal health.

As one of the major post-translational modifications in eukaryotic cells, ubiquitination plays a key role in a wide range of essential biological processes, including immune response regulation, mitochondrial autophagy, DNA damage repair, cell cycle regulation, epigenetic regulation, cell proliferation, apoptosis, and protein degradation. Specifically, ubiquitination influences the stability, activity, and subcellular localization of proteins and alters protein–protein interactions [17]. Dysregulated ubiquitination has been found to cause various diseases, such as cancer, muscular dystrophy, immune disorders, and metabolic syndrome [18]. In recent years, advances in mass spectrometry (MS)-based proteomics have significantly expanded the scope of research on ubiquitination. With the development of techniques that enrich ubiquitinated proteins/peptides, along with the availability of high-throughput, high-coverage, and high-sensitivity mass spectrometry techniques for detecting protein ubiquitination, the field of ubiquitin-specific proteomics has rapidly developed. Today, this technique is being applied to studies on human and animal physiology, pathology, and disease mechanisms. However, the profile of protein ubiquitination in the hepatic tissues of dairy goats with fatty liver remains to be determined.

In this study, we focused on fatty liver in dairy goats to provide a theoretical foundation for diagnosing this disease in ruminants, which is particularly relevant as dairy goat farming continues to grow. We examined protein ubiquitination in dairy goats with fatty liver using liquid chromatography coupled with MS (LC-MS) analysis. We then explored the potential mechanisms involved in the ubiquitination of altered proteins through Kyoto Encyclopedia for Genes and Genomes (KEGG) pathway, Gene Ontology (GO) enrichment, and protein–protein interaction network analysis. Our preliminary findings provide a comprehensive expression profile of ubiquitinated proteins in goat fatty liver, offering insights that could guide the discovery of biomarkers and new therapeutic targets for fatty liver disease in ruminants.

## 2. Materials and Methods

### 2.1. Sample Collection

Six Saanen dairy goats with similar perinatal periods (age ranging from 1 to 2 years, gestation period of 150 days, one fetus per goat) were selected from a goat farm in Inner Mongolia for this study. These animals were classified into two groups based on the results of hepatic hematoxylin and eosin (HE) staining and triglyceride (TG) levels: the fatty liver dairy goat group (FL, *n* = 3) and the healthy dairy goat group (Con, *n* = 3). All the selected dairy goats were fed normally on the goat farm. This study was approved by the Ethics Committee of the Inner Mongolia MinZu University. Liver tissues and blood were collected from the goats after slaughter and stored in liquid nitrogen.

### 2.2. Protein Extraction

Liver tissue samples stored at −80 °C were removed, thawed, and transferred to a homogenizer tube. An appropriate volume of protein extraction buffer, including a phosphatase inhibitor cocktail, was added to the tissue. The samples were homogenized on ice using a tissue grinder (Thermo Fisher Scientific, Wilmington, DE, USA) (3 rounds of 30 min each), with intermittent vortexing (5–10 s every 5 min) to ensure mixing. The homogenized samples were centrifuged at 16,000× *g* and 4 °C for 30 min, and the supernatant containing the soluble proteins was carefully collected. Protein concentrations were determined using the bicinchoninic acid (BCA) assay.

### 2.3. Sodium Dodecyl Sulfate-Polyacrylamide Gel Electrophoresis (SDS-PAGE)

**Figure 1:**
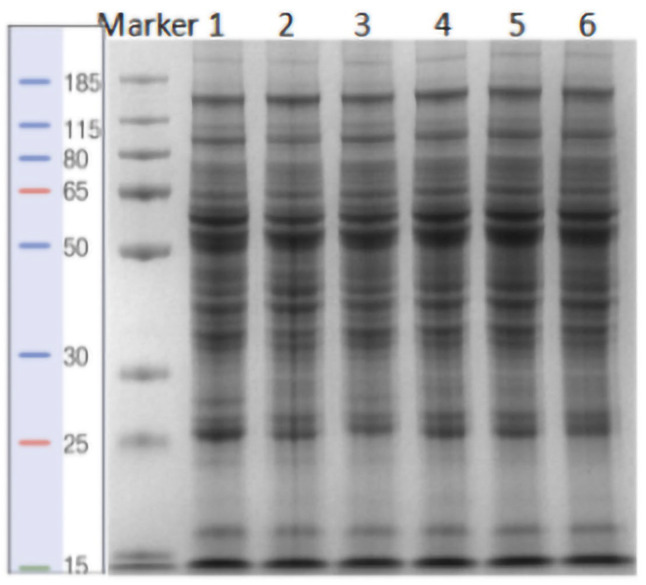
SDS-PAGE gel showing protein bands. Note: Samples 1, 3, and 5: fatty liver dairy goats; samples 2, 4, and 6: healthy dairy goats.

For SDS-PAGE analysis, 15 µg of protein was mixed with an equal volume of 5× SDS loading buffer and heated in a boiling water bath (HWS-12, Shanghai Yiheng Scientific Instrument Co., Ltd., Shanghai, China) for 5 min. Proteins were separated using 12.5% SDS-PAGE (14 mA for 90 min). After electrophoresis, the gel was stained with Coomassie Brilliant Blue to visualize protein bands (Figure 1).

### 2.4. Reductive Alkylation and Enzymatic Hydrolysis

For reductive alkylation, 22 mg of each protein sample was combined with an equal volume of lysis buffer. Then, dithiothreitol (DTT) was added to this mixture (final concentration: 5 mM), and the mixture was incubated at 55 °C for 1 h to reduce disulfide bonds. Subsequently, iodoacetamide (final concentration: 10 mM) was added, and the reaction was allowed to proceed at room temperature for 40 min away from light to alkylate the cysteine residues. The reaction mixture was then diluted four times with 20 mM HEPES buffer. Trypsin was added to the sample at a mass ratio of 1: 100 (enzyme: protein), and digestion was carried out overnight at 37 °C.

### 2.5. Peptide Desalination and Quantification

After enzymatic digestion, the resulting peptides were desalted using Sep-Pak cartridges (Thermo Fisher Scientific, Wilmington, DE, USA) according to the manufacturer’s instructions. For peptide quantification, some of the digested peptides were quantified using the Thermo Fisher Scientific Peptide Quantification Kit (Cat. No. 23275). The remaining peptide sample was freeze-dried using a lyophilizer (Taicang Huamei Biochemical Instrument Factory, Suzhou, China).

### 2.6. Enrichment of Ubiquitinated Peptides

The lyophilized polypeptides were reconstituted in 1× IAP Buffer, and any undissolved material was removed by centrifugation (Eppendorf, Hamburg, Germany). Anti-Lys-ε-Gly-Gly (K-ε-GG) antibody-conjugated beads (Cell Signaling Technology, Danvers, MA, USA), pre-treated by washing four times with 1× PBS, were added to the peptide solution and incubated at 4 °C for 2 h. Following incubation, the beads were pelleted by centrifugation, and the supernatant was removed and frozen. The beads were washed twice with 1× IAP Buffer and three times with LC-MS grade water to remove non-specifically bound peptides. Ubiquitinated peptides were eluted from the beads using 0.15% trifluoroacetic acid (TFA) through two elution steps, with a second round of elution after the initial round of collection. The eluate was centrifuged to remove residual beads. The enriched peptides were then desalted using a StageTip (C18) (Thermo Fisher Scientific, Wilmington, DE, USA) and subjected to vacuum drying.

### 2.7. LC-MS/MS Data Acquisition

Based on the results of peptide segment quantification, peptides (0.5 μg/μL) were dissolved in the mass spectrometry loading buffer. The samples were then dissolved in buffer A (2% acetonitrile, 0.1% formic acid) and loaded onto a C18 column (75 μm × 25 cm, Thermo Fisher Scientific, Wilmington, DE, USA). The peptides were separated using reversed-phase chromatography with a solvent B gradient, as outlined in Table 1. The flow rate was maintained at 300 nL/min using the EASY-nLC 1200 UPLC system (Thermo Fisher Scientific, Wilmington, DE, USA).

Following chromatography, the specimens were introduced into a Q-Exactive HF X tandem mass spectrometer (Thermo Fisher Scientific, Wilmington, DE, USA) for analysis in the data-dependent acquisition (DDA) mode. The instrument parameters were set as follows: MS scan range (*m*/*z*) from 300 to 1500, DDA mode, with the Top 20 strongest parent ions selected for fragmentation. The following mass spectrometry parameters were applied: MS resolution: 60,000, AGC target: 3 × 10^6^, Maximum IT: 50 ms; MS2 resolution: 15,000, AGC target: 5 × 10^5^, Maximum IT: 100 ms; Fixed first mass: 100 *m*/*z*; Minimum AGC target: 4 × 10^3^; Intensity threshold: 4 × 10^4^. Fragment ions were detected by the Orbitrap using the HCD ion fragmentation mode. The dynamic exclusion time was set to 30 s.

### 2.8. Database Search

The software version used for database searching was Proteome DiscovererTM 2.4. The original data file was uploaded to the Proteome Discoverer server for database searching and analysis. The related parameters are shown in Table 2.

### 2.9. Bioinformatics and Statistical Analysis

The differential ubiquitination of peptides between normal liver (Con) versus fatty liver (FL) was determined based on predetermined significance criteria (two-fold up- or down-regulation and *p* < 0.05). The log2 fold-change (log2FC) values of all differentially ubiquitinated peptides corresponding to the same protein were summed to calculate the Δ*p*-value. If no differentially ubiquitinated peptides were detected in a protein, the Δ*p*-value was 0. A Δ*p*-value > 2 indicated the upregulation of ubiquitinated peptides and suggested an increase in the potential ubiquitination level of the protein, while a Δ*p*-value < −2 indicated a downregulation of the ubiquitinated peptide and a decrease in the potential ubiquitination level of the protein. GO annotations and KEGG pathway analysis were performed at the proteomic level using the DAVID database. Ubiquitinated peptide sequences, including the lysine site of the ubiquitinated proteins, were analyzed based on a 13-residue sequence (six amino acids upstream and downstream of the modification site) using Motif analysis software (MEME 5.1.1). The protein sequences obtained from the database were used as backgrounding parameters, and default values were applied for other parameters. Protein–protein interactions and potential functions of the ubiquitinated proteins were further explored using the STRING database and Cytoscape 3.10.1. The results of these bioinformatics analyses were visualized using GraphPad Prism 8.0.2.

### 2.10. Histopathological Examination

Fresh liver samples from goats were fixed in formalin for at least 60 h for histopathological analysis. The formalin-fixed specimens were dehydrated in a gradient of ethanol and xylene solutions. The samples were embedded in paraffin, and liver tissues were sectioned using a Minux S700A slicer (RWD Life Science, Shenzhen, China), mounted on slides, rehydrated with ethanol, and stained with HE. The stained sections were sealed with neutral resin. Histological images were captured using a digital camera mounted on an inverted biomicroscope (Nikon, Tokyo, Japan). Steatosis was assessed by an experienced histopathologist, blinded to animal identification. The histopathologist scored HE-stained hepatocyte sections across multiple fields at ×200 magnification.

### 2.11. Determination of TG Levels in Liver Tissue

A total of 100 mg of unfixed liver tissue was placed in a centrifuge tube and mixed with 0.9 mL of anhydrous ethanol. The tissue was then homogenized in an ice-water bath using an electric homogenizer. Following homogenization, the sample was centrifuged at 4 °C for 10 min at 2500 rpm. After centrifugation, the supernatant was collected and analyzed for the content of TG using the GPO-PAP enzyme method with a TG kit (Nanjing Jiancheng Bioengineering Institute, Nanjing, China).

### 2.12. Determination of Non-Esterified Fatty Acids (NEFA) and β-Hydroxybutyric Acid (BHBA) Levels in the Blood

The concentrations of BHBA and NEFA in blood samples from dairy goats were measured using enzyme-linked immunosorbent assay (ELISA) kits (Nanjing Jiancheng Bioengineering Institute, China).

### 2.13. Quantitative Real-Time PCR Analysis

Total RNA was extracted from goat liver tissue using the TRIzol reagent (Kewin, Jiangsu, China). The RNA was then reverse transcribed into cDNA using the PrimeScriptTM II 1st Strand cDNA Synthesis Kit (TaKaRa, Osaka, Japan). The expression levels of four genes (ACSL1, ACSL5, EHHADH, and ACAA1) were analyzed by RT-qPCR, with Actin serving as the reference gene. RT-qPCR was conducted using cDNA as a template and the FastStart™ Universal SYBR^®^ Green Premix (ROX) (Roche, Basel, Switzerland) according to the manufacturer’s instructions. Data were analyzed using the relative quantification method (2^−ΔΔCt^) based on cycling threshold (Ct) values. The goat gene-specific primers for goat were synthesized by Sangong Bioengineering (Shanghai, China) and are listed in Table 3.

## 3. Results

### 3.1. Histopathological Testing and TG Content Determination

**Figure 2:**
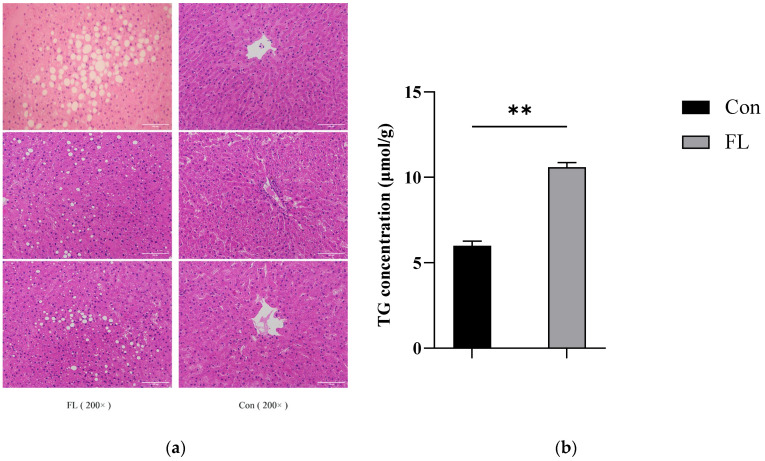
Evaluation of hepatic tissue from goats. (**a**) HE staining images showing representative liver tissue sections. HE staining was performed to assess the pathological changes in the liver. Scale bar = 100 μm. (**b**) Comparison of triglyceride concentrations in the liver between the Con and FL groups. ** *p* < 0.01.

**Figure 3:**
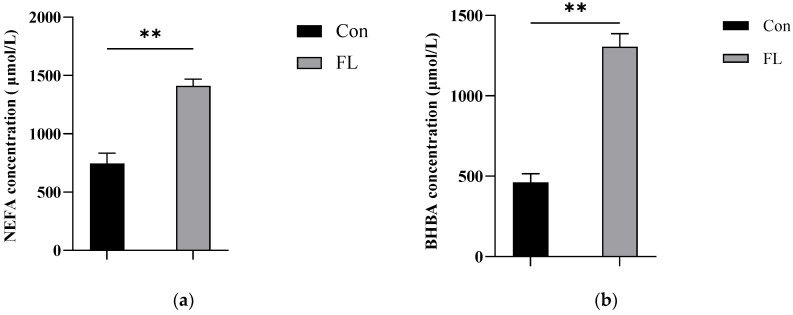
Detection of blood indices in goats. (**a**,**b**) Blood levels of NEFA (**a**) and BHBA (**b**). ** *p* < 0.01.

Liver sections were subjected to HE staining and observed under ×200 magnification (Figure 2a). The concentrations of TG in liver samples (Figure 2b) and that of BHBA and NEFA in blood samples from dairy goats were also examined (Figure 3a,b). The concentrations of TG, BHBA, and NEFA were significantly higher in the FL group than in the Con group. Moreover, the livers of dairy goats from the FL group showed significant steatosis, characterized by large vacuolar steatosis, with hepatocyte nuclei pushed aside by large lipid droplets.

### 3.2. Identification of Ubiquitin-Modified Proteins

**Figure 4:**
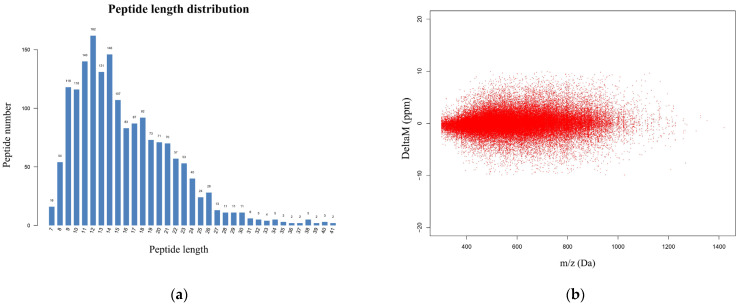

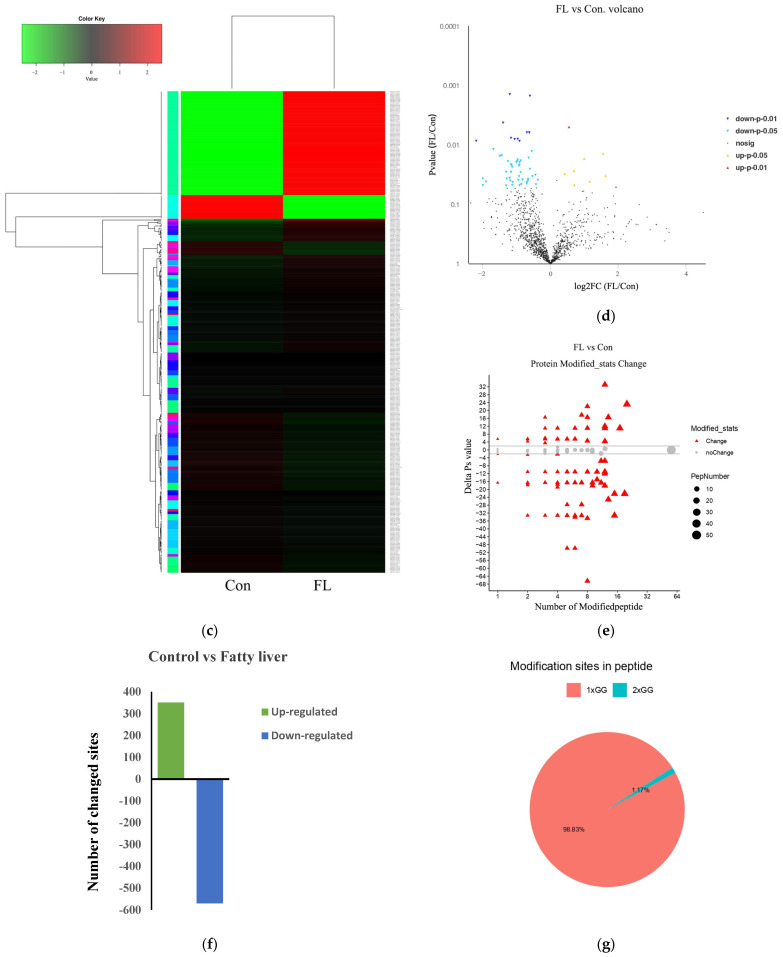
Ubiquitination profiles of hepatic tissue obtained from goats with fatty liver disease. (**a**) Histogram showing the length distribution of identified peptides. (**b**) Distribution of peptide matching errors. (**c**) Heatmap showing differential peptide expression patterns in the Con versus FL groups. (**d**) Volcano plot showing ubiquitylated peptides that differ significantly in expression between the Con versus FL groups. (**e**) Scatter plot of protein ubiquitination status versus peptide number. The grey line indicates the threshold for ubiquitination status. (**f**) Differences in the number of protein ubiquitination sites between the Con and FL groups. (**g**) Distribution of ubiquitination sites in differentially ubiquitinated peptides.

To explore the overall changes in hepatic protein ubiquitination in goats with fatty liver, we performed a comparative analysis of ubiquitination between the FL and Con groups. We used peptide length distributions and mass errors as criteria for validating peptide data. Most of the peptides identified were between 8 and 24 amino acids in length (Figure 4a) and had a mass error of less than 10 ppm (Figure 4b). This validated the high quality and accuracy of our proteomics data, demonstrating its suitability for further analysis.

Heatmap data revealed clear significant differences in protein ubiquitination between the Con and FL groups (Figure 4c). We identified a total of 1871 peptides and 65 differential peptides (9 upregulated and 56 downregulated) (Figure 4d). In total, we identified 1870 ubiquitination sites distributed across 726 host proteins. Of these, 921 ubiquitination sites across 238 proteins were differentially modified between Con and FL tissues. Notably, ubiquitination was upregulated at 351 sites across 93 proteins and downregulated at 570 sites across 145 proteins (Figure 4e,f). Among the ubiquitinated peptides, 98.83% contained one ubiquitination site and 1.17% contained two ubiquitination sites (Figure 4g).

### 3.3. Functional Enrichment of Ubiquitinated Proteins and Protein–Interaction Networks

**Figure 5:**
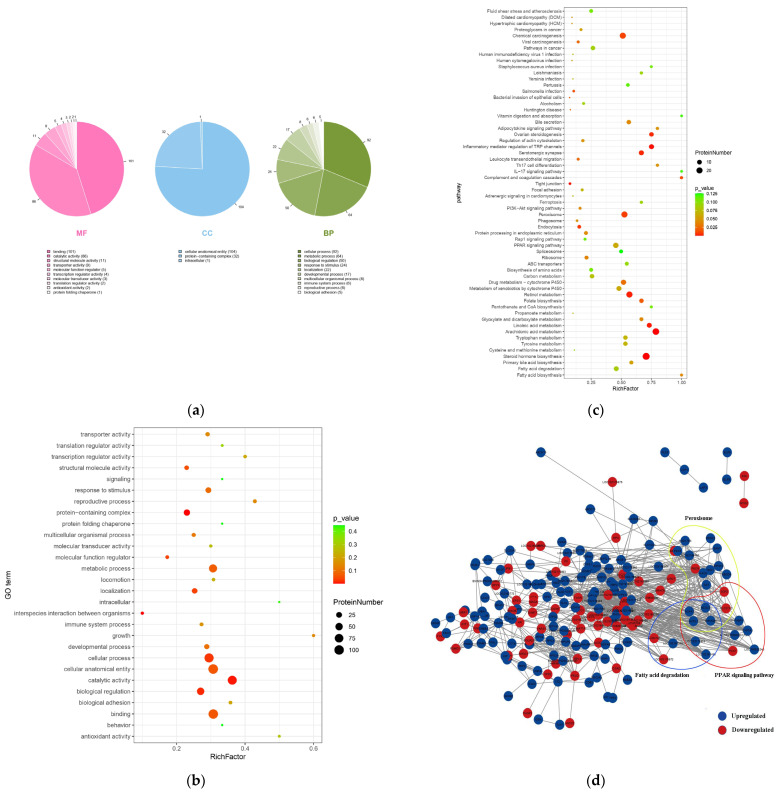
(**a**) Pie chart showing the GO enrichment of differentially ubiquitinylated proteins. (**b**) Bubble diagram showing the GO enrichment of differentially ubiquitinylated proteins. (**c**) Bubble diagram showing the KEGG enrichment of differentially ubiquitinylated proteins. (**d**) Cytoscape software was used to construct the protein–protein interaction network of ubiquitinated proteins. Blue solid circles indicate proteins with downregulated ubiquitinoylation, and red solid circles indicate proteins with upregulated ubiquitinoylation. Peroxisomes: circled by yellow lines; PPAR signaling pathway: circled by red lines; Fatty acid degradation: circled by blue solid lines.

To better understand the changes in lysine ubiquitination in the hepatic tissues of dairy goats with fatty liver, we performed GO functional enrichment analysis. In the cellular component (CC) module, the differentially ubiquitinated proteins were associated with cellular anatomical structures, protein-containing complexes, and intracellular. In the molecular function (MF) module, the differentially ubiquitinated proteins were associated with protein binding, catalytic activity, transporter activity, and other terms. In the biological process (BP) module, the differentially ubiquitinated proteins were associated with cellular processes, metabolic processes, biological regulation, localization, reproductive processes, immune system processes, biological adhesion, locomotion, and other terms (Figure 5a,b). In the MF module, the highest number of differentially ubiquitinated proteins were associated with binding (101 proteins) and catalytic activity (86 proteins). In the BP module, the highest number of differentially ubiquitinated proteins were involved in cellular processes (92 proteins) and metabolic processes (64 proteins).

Advanced bubble diagrams of KEGG pathway enrichment showed that differentially ubiquitinylated proteins were largely enriched in pathways such as chemical carcinogenesis, retinol metabolism, inflammatory mediator regulation of TRP channels, PPAR signaling pathway, fatty acid metabolism, drug metabolism—cytochrome P450, peroxisomes, steroid hormone biosynthesis, and fatty acid degradation (Figure 5c). To further understand the key role of ubiquitination in fatty liver disease, we generated an interaction network using the STRING and Cytoscape databases. We identified three sub-networks regulating the PPAR signaling pathway, fatty acid degradation, and peroxisomes, belonging to the organismal systems, metabolism, and cellular processes categories, respectively. In these three sub-networks, some proteins showed increased levels of ubiquitination, while others showed decreased levels of ubiquitination. By observing the overlap among these three sub-networks, four proteins, including long-chain acyl-CoA synthetase 1 (ACSL1), long-chain acyl-CoA synthetase 5 (ACSL5), enoyl-CoA hydratase/3-hydroxyacyl-CoA dehydrogenase (EHHADH), and acetyl-CoA acyltransferase 1 (ACAA1), were obtained, and all of them were found to show decreased ubiquitination levels (Figure 5d).

### 3.4. Motif Analysis of the Identified and Quantifiable Proteins

To study the regulation of ubiquitination in hepatic tissues of goats with fatty liver disease, ubiquitination motif analysis was performed using Motif-X software. All the 11 base sequences identified are shown in Table 4, and the highest score was obtained for the base sequence xGxxxx_K_Gxxxx (K: ubiquitinated lysine residues; X: arbitrary amino acid residues; and I, G, R, Q, and L: Isoleucine, Glycine, Arginine, Glutamine, and Leucine, respectively).

### 3.5. Further Analysis of Ubiquitination in Hepatic Proteins

Through a comprehensive analysis of ubiquitination data, GO enrichment analysis, and KEGG pathway analysis, four proteins (ACSL1, EHHADH, ACAA1, and ACSL5) were selected for further analysis. Specifically, the ubiquitination levels of all four proteins were found to be significantly lower in the FL group, demonstrating the potential role of the ubiquitination of these four proteins in fatty liver disease. ACSL1 had 8 ubiquitinated peptide segments, with 9 ubiquitination sites (K448, K284, K416, K572, K407, K377, K237, K532, and K243); EHHADH had 15 ubiquitinated peptide segments, with 15 ubiquitination sites (K330, K250, K222, K38, K591, K577, K584, K362, K171, K165, K535, K532, K700, K280, and K318); ACAA1 had 3 ubiquitinated peptide segments, with 3 ubiquitination sites (K395, K292, and K198); and ACSL5 had 3 ubiquitinated peptide segments, with 3 ubiquitination sites (K412, K536, and K616). The abundance of all four groups of ubiquitinated peptide segments was significantly lower in the FL group than in the Con group (Table 5).

**Figure 6:**
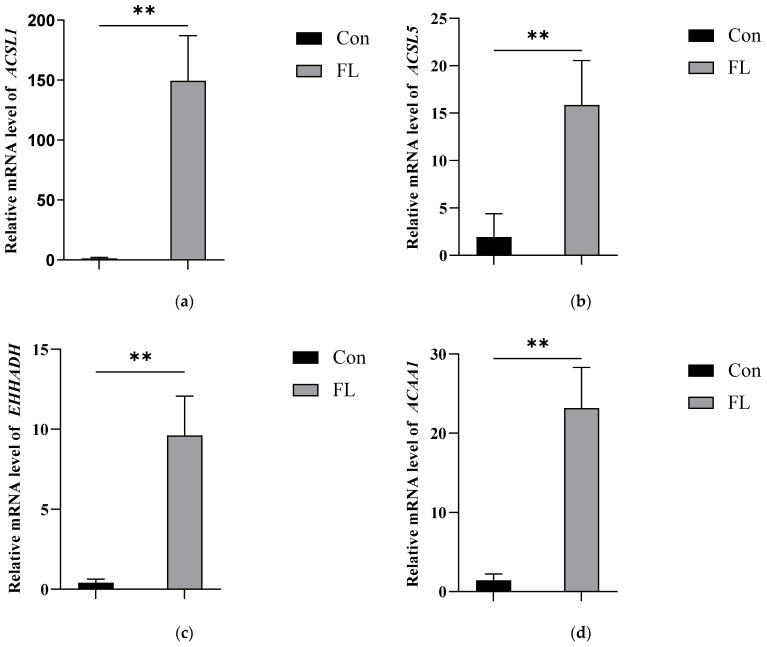
Relative expression of target genes. (**a**–**d**) RT-qPCR analysis of the *ACSL1* (**a**), *ACSL5* (**b**), *EHHADH* (**c**), and *ACAA1* (**d**) genes in the Con and FL groups. ** *p* < 0.01.

We further verified the changes in these four proteins at the transcriptional level using RT-qPCR. The results showed that the four genes (*ACSL1*, *ACSL5*, *EHHADH*, and *ACAA1*) were significantly upregulated in the hepatic tissues of dairy goats with fatty liver (Figure 6). Therefore, the reduced ubiquitination levels in the above four proteins, which are associated with lipid metabolism, appeared to be responsible for their high expression in goats with fatty liver.

## 4. Discussion

The physiological mechanisms associated with fatty liver in dairy goats have so far remained unclear. Most previous research has focused on plasma metabolism in dairy goats with ketosis, and there has been limited research on fatty liver disease in periparturient dairy goats [19]. Evidence from the present study ties the pathogenesis of fatty liver in dairy goats to the accumulation of TG in the liver. In this study, we evaluated goat liver samples using HE staining and TG assays and detected the significant upregulation of blood BHBA and NEFA concentrations in dairy goats with fatty liver. We found that in hepatic tissue from dairy goats with fatty liver, ubiquitination was upregulated at 351 sites across 93 proteins and downregulated at 570 sites across 145 proteins, affecting multiple pathways closely related to lipid metabolism. These findings provide a theoretical basis for the prevention and treatment of fatty liver disease in dairy goats.

Instead of other known methods for analyzing hepatic metabolic dysfunction in ruminants, the present study used ubiquitination proteomics to analyze the protein ubiquitination profile in dairy goats with fatty liver for the first time, elucidating the changes in hepatic proteins under this pathological state. Although ubiquitination is absent in prokaryotes, it is highly conserved in all eukaryotes. The ubiquitin–proteasome system plays a key role in controlling cellular processes in eukaryotes [20,21]. Based on heatmap data (Figure 4c), we found that HECT, UBA, and WWE domain-containing E3 ubiquitin ligase 1 (HUWE1) were differentially ubiquitinated between the Con and FL groups. Several aspects of protein function are regulated by ubiquitination, including protein degradation, transport, and interactions [22]. Through the ubiquitination proteomics profiling of hepatic tissue, in this study, we identified key biological pathways that regulate the pathogenesis of fatty liver in periparturient dairy goats. The analyses showed significant differences in protein ubiquitination between healthy goats and those with fatty liver. KEGG analysis indicated that the differentially ubiquitinated proteins were involved in the PPAR signaling pathway, fatty acid degradation pathway, and peroxisome activity. Hepatic lipid metabolism is regulated by fatty acid uptake and output, and an imbalance leads to hepatic lipid accumulation and fatty liver [23]. Fatty acid metabolism is among the main types of energy metabolism in ruminants and provides energy, raw materials, and biological signals for rapid cell division and proliferation [24]. Approximately 25–50% of the fatty acids in animal tissues are oxidized in peroxisomes, while the others are processed in the mitochondria. In addition, peroxisomes contain enzymes related to phospholipid synthesis, thus contributing to lipid anabolism. The PPAR pathway is thought to play a key role in the pathogenesis of non-alcoholic fatty liver disease (NAFLD), but the underlying mechanisms are not clear [25]. Based on our findings, we speculate that alterations in protein ubiquitination affect the activity of the PPAR signaling pathway in goats, promoting the hepatic accumulation of TG. According to the GO and KEGG pathway analyses, in addition to the above three pathways, hepatic tissues from goats with fatty liver disease also expressed differentially ubiquitinated proteins involved in retinol metabolism, steroid hormone biosynthesis, and arachidonic acid metabolism pathways. This suggests that transcription, protein interactions, and enzyme activities in fatty liver may be modulated through the regulation of protein ubiquitination. Hence, the relevant differentially ubiquitinated proteins identified in this study could be examined for their value treatment targets for fatty liver disease.

By analyzing the interconnections of the PPAR signaling pathway, fatty acid degradation, and peroxisome activity, four proteins with downregulated ubiquitination (ACSL1, ACSL5, EHHADH, and ACAA1) common to these three pathways were systematically examined. Their ubiquitination sites were identified in an attempt to screen for potential therapeutic target sites. In addition to being enriched in the three pathways, ACSL1 and ACSL5, which showed differential ubiquitination levels, were also enriched in pathways related to fatty acid metabolism, ferroptosis, adipocytokine signaling, fatty acid biosynthesis, and thermogenesis. Similarly, EHHADH, which also exhibited altered ubiquitination, was enriched in pathways such as carbon metabolism; tryptophan metabolism; fatty acid metabolism; beta-alanine metabolism; and the degradation of valine, leucine, and isoleucine. Finally, ACAA1 was enriched in fatty acid metabolism; the biosynthesis of unsaturated fatty acids; and the degradation of valine, leucine, and isoleucine.

ACSL1 and ACSL5 can convert fatty acids into long-chain acyl-CoAs and thus play a central role in hepatic lipid metabolism [26]. ACSL1 is a key player in the PPAR signaling pathway and is highly expressed in the tissues involved in energy metabolism [27]. Notably, the levels of ACSL1 influence the lactation performance of dairy cows, including the levels of milk fat and milk protein [28]. ACSL1 can promote the incorporation of conjugated linoleic acid into neutral lipids, causing ferroptosis by increasing lipid peroxidation [29]. The targeted silencing of ACSL1 in mice fed a high-fat diet was found to reduce hepatic ceramide (Cer) and diglyceride (DAG) levels and rescue insulin-stimulated Akt phosphorylation [30]. One study of the ACSL5 gene found that ACSL5 is involved in lipid anabolism, activating and directing fatty acids into anabolic pathways, promoting triglyceride synthesis and lipid droplet storage [31]. In another study, researchers found that the upregulation of the ACSL5 protein in HepG2 cells increased fatty acid oxidation, whereas the knockdown of this protein increased the concentration of TG [32]. Our KEGG pathway analysis showed that EHHADH and ACAA1 are mainly involved in the PPAR signaling pathway, which regulates the cell cycle as well as metabolism [33]. EHHADH and ACAA1 are two of the four enzymes that mediate the peroxisomal β-oxidation pathway and play a crucial role in maintaining normal peroxisome function [34,35]. In the PPAR signaling pathway, EHHADH contributes to fatty acid β-oxidation in a PPAR family member-dependent manner, acting as a catalyst for the degradation of long-chain dicarboxylic acids. Thus, it plays an important role in the peroxisomal fat oxidation pathway in hepatocytes [36]. Accumulating evidence now clearly shows that EHHADH is involved in the metabolism of deoxycholic acid (DCA), which plays an important role in hepatic physiological and pathological processes [37]. ACAA1 is a key enzyme in fatty acid synthesis and transport [38], and its overexpression leads to the enhanced proliferation of mammary epithelial cells [39]. This highlights the important regulatory role of ACAA1 in mammary activity, especially the synthesis of milk components [40]. In a previous study, the transcriptional profiling of Hereford bovine livers revealed that upregulation of the ACAA1 gene was involved in fatty acid biosynthesis and lipid metabolism [41]. This is consistent with the findings of the present study. Collectively, the results suggest that ACSL1, ACSL5, EHHADH, and ACAA1 play an important role in the development of fatty liver disease in dairy goats. Hence, targeting ubiquitination sites in these four proteins could hinder the development of fatty liver disease in these animals.

We speculate that the altered ubiquitination of ACSL1, ACSL5, EHHADH, and ACAA1 contributes to their increased hepatic protein levels and abnormal subcellular localization in dairy goats with fatty liver. Indeed, we observed significantly elevated gene expression of *ACSL1*, *ACSL5*, *EHHADH*, and *ACAA1* in the hepatic tissues of periparturient goats affected by fatty liver disease. Our findings suggest that the reduced ubiquitination of these proteins could be a potential mechanism contributing to fatty liver disease in ruminants. These results provide new perspectives that improve our understanding of fatty liver pathogenesis in dairy goats, which may have important implications for animal husbandry and clinical treatment. Our results suggest that changes in ubiquitination levels are closely associated with the onset and progression of fatty liver. Although fatty liver is currently managed primarily through lifestyle interventions and pharmacological treatments, future studies could explore the development of vaccines that modulate ubiquitination, thereby preventing the onset of fatty liver. Alternatively, by monitoring the ubiquitination levels of relevant proteins, early signs of fatty liver can be detected in a timely manner, and feed formulations and farming environments can be adjusted to reduce the incidence of this condition.

However, given the limited sample size of the current study, further research using a larger number of fatty liver samples is necessary to better elucidate the mechanisms of ubiquitination in this context and to validate our hypothesis. Nevertheless, we ensured the representativeness of our study sample and the reliability of our data through strict data quality control. Our findings provide some basic insights as well as valuable references for the field. In subsequent studies, we plan to collect more samples through multi-center collaboration so as to improve the stability and generalizability of the findings. Meanwhile, we will also explore the mechanisms of ubiquitination in dairy goats with fatty liver in our next study and focus on its potential as a target for managing disease onset, development, and progression.

## 5. Conclusions

In summary, this is the first report to compare the ubiquitination profiles of hepatic proteins between healthy dairy goats and those with fatty liver. The study offers new insights into the diverse cellular processes affected by ubiquitination, which could lead to the identification of better biomarkers and therapeutic targets for fatty liver disease. These findings provide a valuable reference for understanding the biological functions of protein ubiquitination in the context of fatty liver disease in dairy goats.

## Figures and Tables

**Table 1:** EASY-nLC liquid phase gradient.

Time (min)	B (%)
0	5
80	23
99	29
108	35
111	48
112	100
120	Stop

**Table 2:** Search parameters in Proteome DiscovererTM.

Item	Value
ProteomeDiscoverer version	2.4
Protein Database	uniport-taxonomy-9925.unique.fasta
Cys alkylation	Iodoacetamide
Dynamic Midification	Oxidation (M), Acetyl (Protein N-Terminus), Acetyl(K), GG(K, S, T)
Static Modification	Carbamidomethyl (C)
Enzyme Name	Trypsin (Full)
Max. Missed Cleavage Sites	2
Precursor Mass Tolorance	10 ppm

Note: The result filtering parameter was Peptide FDR ≤ 0.01.

**Table 3:** Primer sequence list.

Gene		Primer (5′–3′)
*Capra hircus ACSL5*	F	CGAAAATGGACTCTTGAC
	R	ACTCCTGGGTGTTCTCAT
*Capra hircus ACSL1*	F	GGTTGACTTCCGGCAGTACG
	R	CCAGGTCGCATGGAGGCT
*Capra hircus EHHADH*	F	AGCGTGTCTTTGCTGAAC
	R	AGAATCGGCTGGGAATAA
*Capra hircus ACAA1*	F	TGACGACAAGGGCACAGA
	R	CTTAAAGGCGGGCTTCAG
*Capra hircus Actin*	F	CTCAGAGCAAGAGAGGCAT
	R	CTCGTTGTAGAAGGTGTGGT

**Table 4:** Results of motif enrichment analysis.

Motif	Motif Core	Foreground		Background		Fold Increase
		Matches	Size	Matches	Size	
xGxxxx_K_Gxxxxx	10.55	20	1624	96	22,977	2.9
xxxxxx_K_Gxxxxx	6.07	135	1604	1253	22,881	1.5
xxxGxG_K_xxxxxx	6.89	22	1469	126	21,628	2.6
xxxxxx_K_xxxxxR	3.64	106	1447	1106	21,502	1.4
xxxxxx_K_xxxRxx	3.13	91	1341	91	20,396	1.4
xxxxxx_K_xxGxxx	2.72	108	1250	108	19,418	1.3
xxxxIx_K_xxxxxx	2.58	85	1142	85	18,156	1.4
xxxxxL_K_xxxxxx	3.13	147	1057	147	17,168	1.3
xxGxxx_K_xxxxxx	3.22	75	910	75	15,326	1.5
xxIxxx_K_xxxxxx	2.38	60	835	60	14,471	1.4
xQxxxx_K_xxxxxx	2.21	49	775	49	13,743	1.5

**Table 5:** Differences in the ubiquitination of key proteins between the FL and Con groups.

Protein	PepNumber	Delta *p*-Value (FL/Con)	Ubiquitination Sites
ACSL1	8	−34.62495976	K448; K284; K416; K572; K407; K377; K237; K532; K243
EHHADH	15	−22.19208675	K330; K250; K222; K38; K591; K577; K584; K362; K171; K165; K535; K532; K700; K280; K318
ACAA1	3	−16.61	K395; K292; K198
ACSL5	3	−16.61	K412; K536; K616

## Data Availability

The data presented in this study are available upon reasonable request from the corresponding author as they are currently being used in ongoing research projects.
